# Expanding ultrasound horizons in inflammatory bowel disease: Beyond the bowel

**DOI:** 10.1002/ueg2.12580

**Published:** 2024-05-08

**Authors:** Paula Sousa, Cláudio Rodrigues

**Affiliations:** ^1^ Department of Gastroenterology of Hospital São Teotónio – Unidade Local de Saúde Viseu‐Dão Lafões Viseu Portugal

**Keywords:** Inflammatory Bowel Disease, Sarcopenia, Ultrasound

Sarcopenia is a syndrome of increasing importance due to its increasing prevalence and significant personal, social and economic impact.^1^, ^2^ Recent definitions characterize it as a syndrome marked by reductions in both skeletal muscle mass and strength.^2^ Although conventionally linked with the elderly, sarcopenia can also occur in younger subjects secondary to other pathologic conditions, as is the case of inflammatory bowel disease (IBD).^3^, ^4^ In IBD, sarcopenia is associated with adverse clinical outcomes including heightened risk of treatment failure, age‐related mortality, and post‐operative complications.^3^, ^4^

Health implications of sarcopenia underscore the necessity for screening and early detection. While various methods exist—such as magnetic resonance imaging (MRI), computed tomography (CT), dual‐energy X‐ray absorptiometry (DXA) and bioelectrical impedance analysis (BIA)—limitations impede their routine use. The ideal tool should be easily accessible, cost‐effective, and reproducible across different clinical settings. Ultrasound is emerging as an alternative for the detection of sarcopenia, as it is a bedside tool that assesses both muscle quantity and quality. It has been proven to be accurate in the estimation of muscle mass in comparison to MRI, CT and DXA in the general population.^2^, ^3^, ^5^ Additionally, it has been demonstrated to reliably assess sarcopenia in the elderly and in different comorbid populations, where it was associated with the occurrence of clinical outcomes.^6^, ^7^, ^8^

In this issue of the United European Gastroenterology Journal, Mulinacci and colleagues evaluated the potential of muscle ultrasound as a diagnostic tool of sarcopenia in IBD patients.^9^ Measurements were performed in three superficial muscles—biceps brachii, rectus femoris and the rectus abdominis—in alignment with SARCUS working group recommendations.^5^ Initially, the feasibility and reliability of ultrasound measurements was evaluated in a training cohort comprised of 100 patients (56 of which with IBD). The inter‐ and intra‐operator agreement for ultrasound measurements was excellent, suggesting that ultrasound is a reliable and reproducible tool, if clinicians follow a clear protocol. The accuracy of ultrasound in the detection of sarcopenia was subsequently tested in a cohort of 53 IBD patients using BIA as the reference standard, with the demonstration of good accuracies, with AUROC ranging from 76% to 85%. Two innovations of this study were the development of an Ultrasound Muscle Index, with a good accuracy when compared to BIA (AUROC of 81%), and the establishment of optimal cutoff values for ultrasound measurements in diagnosing sarcopenia in IBD patients.

While the study offers significant insights, it has some limitations, including a small sample size and the use of BIA cutoffs from elderly Asian populations, which may not be directly applicable to this population. Indeed, studies on sarcopenia in the IBD population are hindered by the lack of established cut‐off for muscle mass and function in this population.^3^ Additionally, only muscle quantitative parameters were measured. Still, this study represents a crucial advancement in sarcopenia assessment in IBD and, to our knowledge, it's the first assessing the use of ultrasound in this context.

As demonstrated in this study, sarcopenia is a prevalent condition in IBD patients. Considering that interventions to improve muscle health are relatively simple and economic, a proactive approach to enhance awareness among physicians and patients is warranted. It should be emphasized that sarcopenia can affect patients in a wide range of body mass indices, from underweight to obese, and, therefore, screening should be considered in all IBD patients.^3^, ^10^ In this regard, ultrasound offers clear advantages in relation to other methods, namely its accessibility, non‐invasiveness, portability, and safety. Moreover, intestinal ultrasound is becoming an integral part of monitoring in IBD, allowing for assessment of disease activity, extension, and response to treatment.^11^ The widespread use of ultrasound in IBD provides an opportunity for clinicians to assess muscle health during the same procedure.

However, there are still areas of uncertainty and challenges that should be addressed before the routine use of ultrasound as a diagnostic tool for sarcopenia. First, intestinal ultrasound is primarily performed by gastroenterologists and IBD specialists, which if, in one hand, allows for timely therapeutic intervention, on the other hand means that specific training for muscle evaluation will be needed.^12^ Furthermore, the technique should be clearly standardized and cutoff values for normal muscle mass and function should be validated across different populations. Other techniques such as ultrasound elastography and contrast‐enhanced ultrasound should be the subject of further research. Finally, prospective studies with serial assessment of muscle health and disease activity should be conducted to demonstrate the impact of the latter in the development of sarcopenia, and to test different therapeutic interventions.

## CONFLICT OF INTEREST STATEMENT

The authors declare no conflicts of interest related to products discussed in the editorial.
